# Modelling human connectome development: precursors to neural circuits

**DOI:** 10.1186/1471-2202-12-S1-P163

**Published:** 2011-07-18

**Authors:** Sreedevi Varier, Marcus Kaiser

**Affiliations:** 1 School of Computing Science, Newcastle University, UK; 2 Institute of Neuroscience, Newcastle University, UK; 3 Department of Brain and Cognitive Sciences, Seoul National University, Korea

## 

There are several brain disorders like schizophrenia, autism and certain kinds of epilepsy, which have their origins in early brain development. *In vivo* studies can be extremely complex and riddled with technical and practical hurdles, heralding the need for *in silico* models for testing hypotheses. To fully understand brain network diseases, it is essential to find out which developmental factors lead to altered network topologies and resulting functional changes such as waves or large-scale activations (as for epileptic seizures). Recent results on local neuronal development have shown how preferences for short-distance connections [1] as well as how long-distance connections can arise [2].

Here, we present a model that simulates the layered development of the human neocortex, from the division of neural precursors to the establishment of layers and connections. Results from the model focus on the emerging network characteristics of cortical networks as synaptic connections are established. We test how quantitative and qualitative network features relate to changes in developmental factors, such as time-windows, spatial positions and chemical gradients.

Results from the model include identification of developmental factors influential in the formation of cortical networks, highlighting those yielding aberrations. In conclusion, the model might inform future clinical and experimental work on the developmental origins of brain network disorders.
